# Association between free triiodothyronine, resting metabolic rate, systemic immune-inflammation index levels and diabetic retinopathy in type 2 diabetic patients with euthyroidism

**DOI:** 10.3389/fendo.2026.1777977

**Published:** 2026-03-27

**Authors:** Min Li, Xinxin Li, Meimei Tian, Pingping Lou, Zhixing Wu, Xi Wang, Huijie Ma, Yan Liu, Xinli Jiang

**Affiliations:** 1 Department of Endocrinology, Hebei Medical University Third Hospital, Shijiazhuang, Hebei, China; 2 Department of Physiology, School of Basic Medicine, Hebei Medical University, Shijiazhuang, Hebei, China; 3 Department of Ophthalmology, Hebei Medical University Third Hospital, Shijiazhuang, Hebei, China

**Keywords:** diabetic retinopathy, euthyroidism, free triiodothyronine, resting metabolic rate, systemic immune-inflammation index

## Abstract

**Introduction:**

Thyroid hormones (TH) play important role in retinal development and maintaining retinal functional hemostasis. Resting metabolic rate (RMR) provides an estimate of the minimum amount of energy expenditure and has a bearing on insulin resistance. Systemic immune-inflammation index (SII) is a new inflammatory biomarker associated with diabetic complications. Inflammation and energy metabolism imbalance are closely related to the pathogenesis of diabetes retinopathy (DR). However, few studies have investigated the relationship between TH, RMR, SII and DR in type 2 diabetic patients (T2DM) with euthyroidism. This study aims to investigate the association of TH, RMR and SII with DR in Chinese T2DM patients with euthyroidism.

**Methods:**

This cross-sectional study was performed in 819 T2DM patients with euthyroidism. Patients were divided into NDR group (n=609) and DR group (n=210). Baseline data and biochemical parameters of patients were collected. Spearman’s correlation analysis, receiver operating characteristic (ROC) curve analysis and logistic regression analysis were performed to examine the association between TH, RMR, SII and DR.

**Results:**

Compared with NDR patients, the FT3 levels were significantly lower in DR patients, no differences were found in FT4 and TSH levels between the two groups. Furthermore, the SII levels were significantly higher while RMR were lower in DR patients. In multivariate analysis, FT3 and RMR were inversely while SII were positively correlated with the occurrence of DR in T2DM patients with euthyroidism. After adjusting for age and sex, fourth quartile of FT3 showed significantly decreased OR of 0.615 while SII showed increased OR of 1.660 for DR with respect to its first quartile value. In the fully adjusted models, fourth quartile of RMR showed significantly decreased OR of 0.291 for DR with respect to its first quartile value.

**Conclusion:**

In euthyroidism patients with T2DM, decreased FT3, RMR and increased SII were correlated with the presence of DR.

## Introduction

Diabetic retinopathy (DR) is one of the most common microvascular complications of diabetes mellitus and remains a leading cause of loss of vision and blindness globally (1). Worldwide, the prevalence of DR has been predicted to be 35% and the prevalence of vision-impairing is estimated around 10% (2). Moreover, as diabetes itself is reaching an epidemic level, DR patients numbers will continue to escalate (3). Recently substantial scientific evidences have confirmed that early diagnosis and timely treatment can prevent most visual loss from DR (4). However, the understanding of DR pathophysiology remains unclear, and current diagnostic methodology is only semi-quantitative and categorizes different fundus observations into non-proliferative retinopathy, diabetic macular edema, or proliferative diabetic retinopathy, which leaves approximately 50% of diabetes patients missed diagnosis (3). Hence, it is of the utmost urgency to identify potential modifiable risk factors that can be quantified and serve to determine the pathogenesis and detect DR at an early stage.

Thyroid hormones (THs) have a significant effect on metabolic processes. Lower levels of serum free triiodothyronine (FT3), even in the euthyroid state, can result in elevated blood glucose and dyslipidemia, thus increasing the risk of diabetic nephropathy (DN) (5) and diabetic peripheral neuropathy (DPN) (6). TH is essential in retinal development and functional maintaining (7). The correlations of TH with DR have been studied previously, in which hypothyroidism (8) and subclinical hypothyroidism (9) are both found related to the presence of DR. Recently, the impact of TH within the normal range on DR has gained much attention, in which the conclusions were quite inconsistent (10, 11).

Obesity has long been recognized as a key predictor for DR due to subclinical inflammation and cellular oxidative stress (12). However, recent studies have generated conflicting results with greater body mass index (BMI) being related to a higher risk of DR (13) or a lower risk of DR (14). The essence of obesity is an imbalance in energy metabolism. Is it obesity itself, or the specific metabolic state accompanying obesity, that really affects the progress of DR? Resting metabolic rate (RMR) represents the minimum energy required to maintain an organism and accounts for 60-70% of total energy expenditure in most individuals. It is the core of an individual’s basal energy expenditure and can more accurately reflect intrinsic metabolic efficiency than BMI. Furthermore, RMR is also reported to be associated with insulin resistance (15), which serve as an risk factor for DR. Therefore, the role of RMR on DR deserves further investigation.

Leukocyte and its subtypes are recognized as the biomarkers of inflammatory response (16). Systemic immune-inflammation index (SII), a combination of three peripheral blood inflammatory cells (neutrophils, lymphocytes and platelets), has been widely investigated as a prognostic indicator in many different clinical diseases, such as cancers, coronary artery diseases, cerebrovascular events and autoimmune disorders (17, 18). Recently, elevated SII value has also been found strongly associated with some ophthalmological diseased such as primary open angle glaucoma (19), dry eye (20), diabetic macular oedema (17) and DR (21). Nevertheless, the relationship between SII and DR in Chinese populations has been little checked, and the present results are somewhat contradictory. Increased SII levels have been found in PDR patients by existing research, however either increased (22) or no altered SII levels (23) were discovered in NPDR patients.

TH, as the core hormones that regulate human metabolism, can affect RMR by promoting cellular oxidative metabolism, and also have regulatory effect on inflammatory responses. However, there is no research on the correlation between TH, RMR, SII and DR. Therefore, the present study aimed to investigate the association of three factors with the presence of DR and to explore their potential as markers of DR in T2DM patients with euthyroidism.

## Materials and methods

### Study design and participants

**Figure 1:**
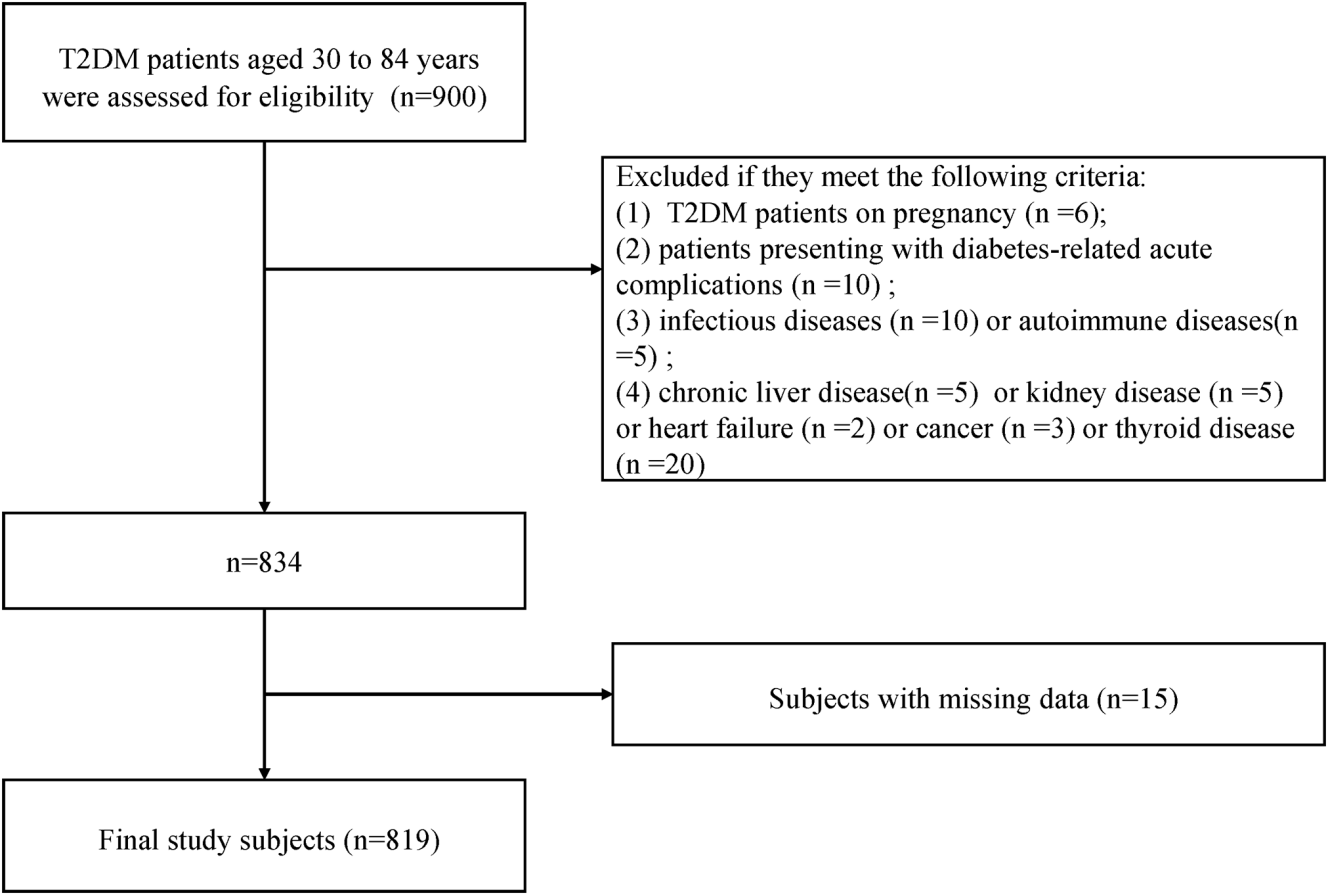
Flow diagram of participant enrollment.

This cross-sectional study was carried out in the Department of Endocrinology, Hebei Medical University Third Hospital from January 2025 to November 2025. The minimum sample size of 378 was calculated using t-tests in G*Power statistical analysis software version 3.1.9.7 considering an effect size of 0.5, alpha error probability of 0.05, power of 0.95, and allocation ratio of N1/N2 of 2, having a sample of 79 DR and 157 NDR. A total of 900 subjects were included based on the following criteria: (1) T2DM diagnosis according to WHO (24); (2) age ranged from 30 to 84 years. We excluded 130 participants who could meet the following criteria: (1) T2DM patients on pregnancy (n=6); (2) patients presenting with diabetes-related acute complications, such as diabetic ketoacidosis, hyperglycemic hyperosmolar state, and lactic acidosis, or having comorbidities such as infectious diseases, autoimmune diseases, malignancy, heart failure, thyroid disease, sever renal and hepatic functional impairment (n=60); (3) patients with missing data (n=15) (**Figure 1**).

Ophthalmologic examination was performed and slit lamp biomicroscopy and retinopathy status was evaluated by fundus photography, fluorescein angiography and optical coherence tomography. DR was diagnosed by a professional ophthalmologist in accordance with the Early Treatment of Diabetic Retinopathy Study (ETDRS) scale (25). T2DM patients were divided into non-diabetic retinopathy group (NDR) and diabetic retinopathy group (DR). Normal reference ranges of TH were: FT3 3.53~7.37pmol/l, FT4 7.98~16.02pmol/l, TSH 0.56~5.91uIU/ml.

Finally, there were total 819 T2DM patients of age ranged 30–84 years enrolled based on the inclusion and exclusion criteria, including 609 NDR patients and 210 DR patients.

This study was carried out in accordance with the Declaration of Helsinki and approved by Ethics Committee of Hebei Medical University Third Hospital (No.: W2025-055-1). Written informed consents were signed by all participants.

### Clinical data and blood samples collection

Baseline data of patients, including age, gender, height, weight, systolic blood pressure (SBP), diastolic blood pressure (DBP), duration of diabetes, antidiabetic drugs, family history and history of smoking and drinking were recorded. Body mass index (BMI) was calculated as weight (kg)/height in meters squared (m^2^). Estimating RMR was calculated using the Mifflin-St Jeo formula (kcal/d). RMR (males) = 10 x weight (kg) + 6.25 x height (cm) - 5 x age (y) + 5; RMR (females) = 10 x weight (kg) + 6.25 x height (cm) - 5 x age (y) - 161.

Total 5ml fasting blood samples were collected. Levels of complete blood count, triglycerides (TG), total cholesterol (TC), low density lipoprotein cholesterol (LDL-C), high density lipoprotein cholesterol (HDL-C), alanine aminotransferase (ALT), aspartate aminotransferase (AST), TBIL (total bilirubin), DBIL (direct bilirubin); creatinine (Cr), fasting C peptide (FC-P), fasting plasma glucose (FPG), hemoglobin A1c (HbA1c), free triiodothyronine (FT3), free thyroxine (FT4) and thyroid stimulating hormone (TSH) were measured in the biochemical laboratory. The estimated glomerular filtration rate (eGFR) was calculated using the Chronic Kidney Disease Epidemiology Collaboration equation. The SII value was calculated as follows: platelet count × (neutrophil/lymphocyte).

### Statistical analysis

All statistical analyses were performed using SPSS software (version 26), and graphs were made using GraphPad Prism 8.0 software. Normally distributed parameters were expressed as mean ± standard deviation (mean ± SD) and compared by independent sample t-test. Non-normally distributed data were expressed as median and interquartile range (IQR) and compared using Mann-Whitney U tests. Categorical variables were expressed as numbers and compared using the chi-square test. Spearman’s correlation analysis was performed to evaluate the correlations between the study factors and the clinical and biochemical parameters. Multivariate logistic regression analyses were performed to detect the contribution of FT3, RMR and SII on the presence of DR. Receiver operating characteristic (ROC) curve analyses were done to evaluate the diagnostic value of parameters for DR. The best cut-off values were calculated using the Youden index, which were calculated according to the corresponding sensitivity, specificity for each index. A two-sided P value <0.05 were considered statistically significant.

## Results

### Demographic and clinical characteristics of the study participants

Data from this study has indicated that, there were no differences in other biochemical parameters including gender, BMI, DBP, history of tobacco and alcohol, DBIL, FPG, HbA1c, Cr and lipid profiles between the two groups (all P>0.05). Compared with NDR patients, patients with DR had a significant increase in age, SBP, duration of diabetes and insulin usage while a decrease in ALT, AST, TBIL, eGFR, and FC-P (all P<0.05). In addition, the SII levels were significantly higher, while RMR were lower in the patients with DR than in those without DR (P<0.05). Meanwhile, patients with DR had lower FT3, but there were no significant differences in FT4 and TSH levels between the two groups(P>0.05) (**Table 1**).

**Table 1:** Demographic, clinical and biochemical characteristics among study subjects.

Characteristic	NDR (n = 609)	DR (n = 210)	P value
Age (year)	59 (49, 67)	60 (53, 67)	0.027*
Gender (female/male)	211/398	77/133	0.597
Duration (years)	8.70 ± 7.94	14.49 ± 8.02	<0.001*
BMI (kg/m^2^)	26.48 ± 3.77	26.13 ± 3.71	0.248
SBP (mmHg)	132.41 ± 15.95	136.03 ± 16.03	0.005*
DBP (mmHg)	85.93 ± 11.28	86.34 ± 10.95	0.645
Family history (yes/no)	321/288	112/98	0.876
Smoking habit (yes/no)	237/372	74/136	0.344
Drinking alcohol (yes/no)	76/533	25/185	0.827
Insulin (yes/no)	181/428	118/92	<0.001*
FPG (mmol/L)	9.03 ± 3.73	8.67 ± 3.60	0.229
HbA1c (%)	8.48 ± 1.94	8.65 ± 1.80	0.226
TC (mmol/L)	4.70 ± 1.34	4.73 ± 1.97	0.834
TG (mmol/L)	1.54 (1.04, 2.31)	1.39 (0.92, 2.09)	0.805
LDL-C (mmol/L)	2.93 ± 1.04	2.86 ± 1.04	0.468
HDL-C (mmol/L)	1.13 ± 0.31	1.16 ± 0.29	0.417
ALT (U/L)	22 (15, 34.1)	19 (15, 27)	<0.001*
AST (U/L)	19.2 (15.35, 24.9)	18.05 (15, 22)	<0.001*
TBIL (umol/L)	14.1 (10.7, 17.95)	13.45 (10.5, 16.13)	0.017*
DBIL (umol/L)	4.00 ± 1.77	3.76 ± 1.56	0.08
Cr (umol/L)	69.71 ± 21.64	72.91 ± 43.84	0.334
eGFR (ml/min)	97.89 ± 18.78	94.70 ± 19.25	0.035*
FC-P (ng/mL)	2.60 ± 1.43	2.05 ± 1.40	<0.001*
TSH (uIU/ml)	2.11 ± 1.11	1.95 ± 0.99	0.063
FT4 (pmol/l)	11.23 ± 1.67	11.26 ± 1.66	0.791
FT3 (pmol/l)	5.02 ± 0.66	4.83 ± 0.63	<0.001*
SII	412.75 (309.63, 577.08)	486.08 (339.13, 679.11)	0.001*
RMR (kcal/d)	1475.03 ± 269.53	1426.29 ± 260.88	0.023*

Data presented as mean ± standard deviation or median (inter-quartile range).

NDR, non-diabetic retinopathy; DR, diabetes retinopathy; BMI, body mass index; HbA1c, hemoglobin A1c; TC, total cholesterol; TG, triglycerides; HDL-C, high density lipoprotein cholesterol; LDL-C, low density lipoprotein cholesterol; ALT, alanine aminotransferase; AST, aspartate aminotransferase; TBIL, total bilirubin; DBIL, direct bilirubin; Cr, creatinine; eGFR, estimated glomerular filtration rate; FC-P, fasting C peptide; FT3, free triiodothyronine; FT4, free thyroxine; TSH, thyroid stimulating hormone; RMR, resting metabolic rate. *Statistically significant (p < 0.05).

### Prevalence of DR according to quartiles of FT3, RMR and SII

**Figure 2:**
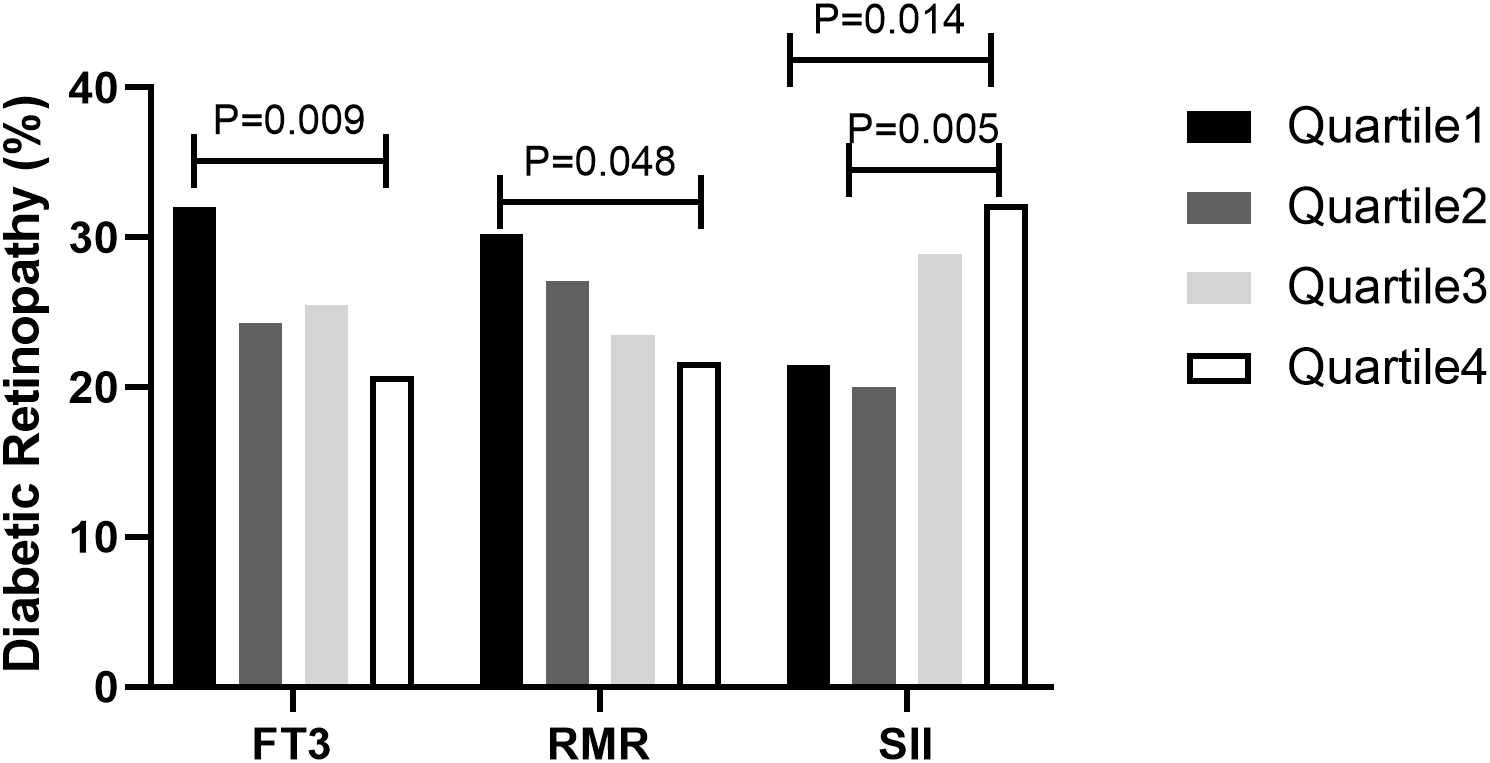
The prevalence of diabetic retinopathy (DR) by quartiles of FT3, RMR and SII in T2DM patients with euthyroidism. FT3, free triiodothyronine; RMR, resting metabolic rate; SII, systemic immune-inflammation index. FT3, P trend=0.016; RMR, P trend=0.033; SII, P trend=0.003.

The FT3, RMR and SII were divided into four groups according to quartiles, respectively. As shown in **Figure 2**, the prevalence of DR was 32.0, 24.3, 25.5, and 20.7% in the first, second, third, and fourth quartile of FT3, respectively. Meanwhile, the prevalence of DR was 30.2, 27.1, 23.5, and 21.7% in the first, second, third, and fourth quartile of RMR, respectively. Furthermore, the prevalence of DR was 21.5, 20.0, 28.9, and 32.2% in the first, second, third, and fourth quartile of SII, respectively. Compared to the first quartile, patients in the fourth quartile for FT3 exhibited a notably lower incidence rate of DR, whereas those in the fourth quartile for SII demonstrated a significantly higher incidence rate of the condition with respect to its second quartile (P < 0.0125) (**Figure 2**).

### Correlation analysis of FT3, RMR and SII

In T2DM patients with euthyroidism, serum FT3 was positively correlated with RMR (r=0.346, P <0.001), BMI (r=0.346, P <0.001), HbA1c (r=0.096, P = 0.006), LDL-C (r=0.123, P <0.001), ALT (r=0.245, P <0.001), eGFR (r=0.283, P <0.001), and FC-P (r=0.245, P < 0.001), while negatively correlated with age (r=-0.373, P <0.001) and duration (r=-0.246, P<0.001), respectively. SII was positively correlated with age (r=0.133, P <0.001), SBP (r=0.128, P <0.001) and duration (r=0.142, P<0.001), while negatively correlated with RMR (r= -0.091, P = 0.009), ALT (r= -0.134, P <0.001) and eGFR (r= -0.131, P <0.001), respectively. RMR was positively correlated with BMI (r=0.561, P <0.001), HbA1c (r=0.102, P = 0.004), ALT (r=0.257, P<0.001), eGFR (r=0.314, P <0.001), and FC-P (r=0.263, P<0.001), while negatively correlated with age (r=-0.558, P<0.001) and duration (r=-0.268, P<0.001), respectively. However, no correlation was found between FT3 and SII levels (r=0.052, P = 0.136) (**Table 2**).

**Table 2:** Correlation analysis of circulating FT3, RMR and SII.

Variable	FT3		RMR		SII	
	r	P	r	P	r	P
age	-0.373	<0.001*	-0.558	<0.001*	0.133	<0.001*
duration	-0.246	<0.001*	-0.268	<0.001*	0.142	<0.001*
RMR	0.346	<0.001*	1		-0.091	0.009*
SII	0.052	0.136	-0.091	0.009*	1	
FT3	1		0.346	<0.001*	0.052	0.136
BMI	0.346	<0.001*	0.561	<0.001*	-0.006	0.854
FC-P (ng/mL)	0.245	<0.001*	0.263	<0.001*	0.001	0.970
SBP	-0.026	0.463	-0.019	0.578	0.128	<0.001*
HbA1c	0.096	0.006*	0.102	0.004*	-0.016	0.655
LDL-C	0.123	<0.001*	0.041	0.240	-0.026	0.458
ALT	0.245	<0.001*	0.257	<0.001*	-0.134	<0.001*
eGFR	0.283	<0.001*	0.314	<0.001*	-0.131	<0.001*

Data presented as correlation coefficient (r). Abbreviations as **Table 1**. *Statistically significant (p < 0.05).

### Receiver operating characteristic curve analysis

**Figure 3:**
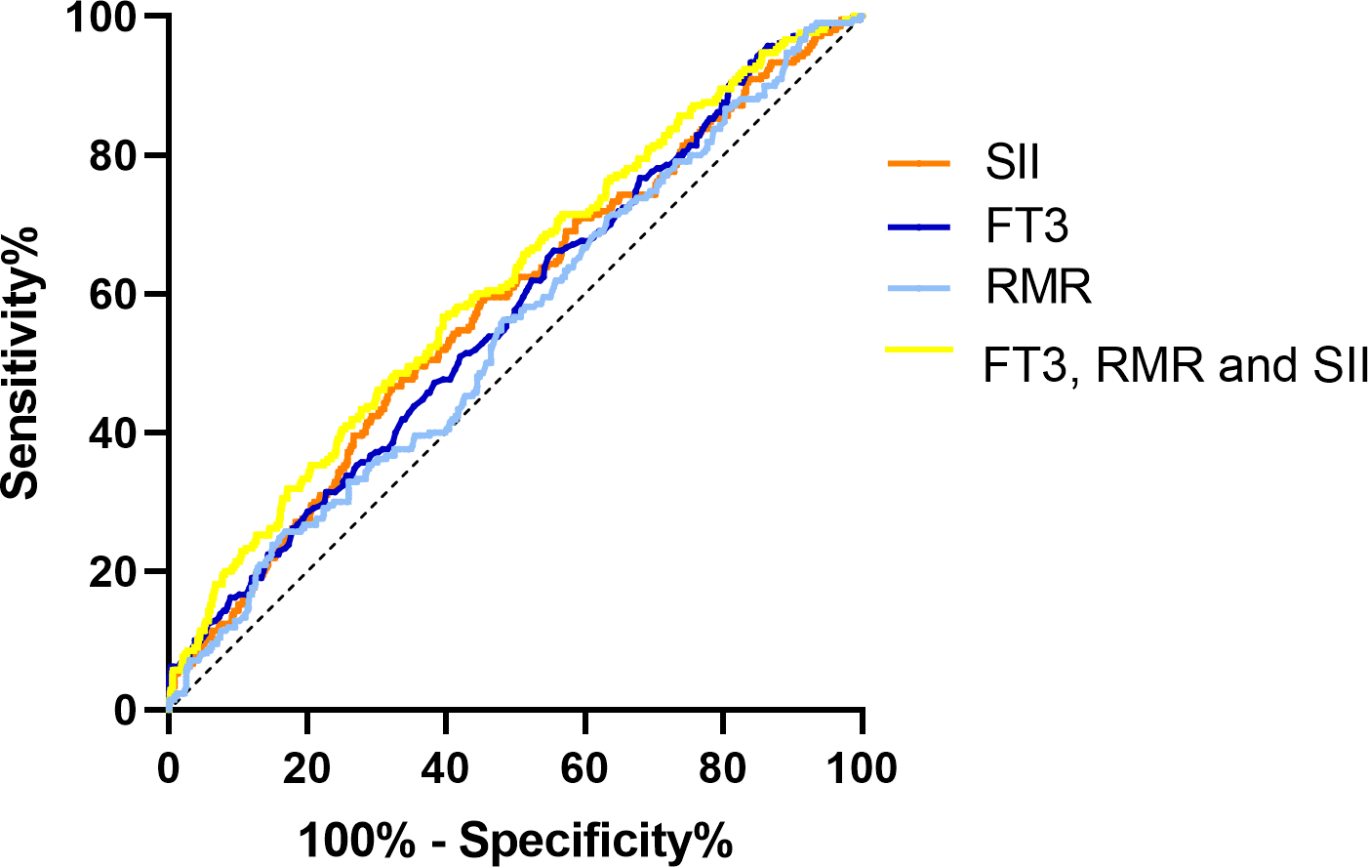
Diagnostic performance of FT3, RMR and SII in identifying diabetic retinopathy. FT3, free triiodothyronine; RMR, resting metabolic rate; SII, systemic immune-inflammation index; ROC, receiver operating characteristic; AUC, area under curve.

ROC curves were performed to check the diagnostic value of FT3, RMR and SII for DR. According to the ROC curve shown in **Figure 3**, the area under the curve (AUC) for FT3 in the diagnosis of DR was 0.571 (95% confidence interval [CI]: 0.526 to 0.615; P = 0.002), and at a cut-off point set at 5.06pmol/l, the sensitivity was 66.2% and specificity was 44.5%. The AUC for RMR was 0.547 (95% CI: 0.502 to 0.592; P = 0.042), and at a cut-off point set at 1180.25, the sensitivity was 25.7% and specificity was 83.3%. The AUC for SII was 0.579 (95% CI: 0.534 to 0.624; P = 0.001), and at a cut-off point set at 522.91, the sensitivity was 46.7% and specificity was 67.8%. In addition, the AUC of FT3, RMR and SII combination was 0.612 (95% CI: 0.568 to 0.656; P = 0.023), yet it did not demonstrate superior predictive value compared to any single indicator.

### Association of DR with FT3, RMR and SII

Multivariate logistic regression analysis was done to assess the associations of FT3, RMR and SII with the presence of DR. As shown in **Table 3**, there were distinct associations of decreased FT3, RMR and increased SII with DR, after adjusting for variables including age, sex, BMI, duration, SBP, HbA1c, LDL-C, ALT and eGFR (P<0.05). Taking the NDR group as the reference category, serum FT3 and RMR were found to be protective factors for DR (odds ratio [OR]0.741; 95% CI: 0.552 to 0.994; P = 0.045 and OR:0.997; 95% CI: 0.995 to 0.999; P = 0.002, respectively) while SII was a risk factor for DR (OR:1.001; 95% CI: 1 to 1.001; P = 0.002) in the fully adjusted model.

**Table 3:** Multivariate logistic regression analysis of the independent factors for presence of DR (FT3, RMR and SII as continuous variable).

Variable	Model 1		Model 2		Model 3	
	OR (95%CI)	P value	OR (95%CI)	P value	OR (95%CI)	P value
FT3	0.623 (0.484-0.802)	<0.001*	0.644 (0.491-0.845)	0.001*	0.741 (0.552-0.994)	0.045*
RMR	0.999	0.023*	0.999	0.039*	0.997	0.002*
	(0.999-1.000)		(0.998-1.000)		(0.995-0.999)	
SII	1.001 (1.001-1.002)	<0.001*	1.001 (1.000-1.002)	<0.001*	1.001 (1.000-1.001)	0.002*

Model 1, not adjusted for any variable; Model 2, adjusted for age, sex; Model 3, adjusted for model 2, BMI, duration of diabetes, SBP, HbA1c, LDL-C, ALT and eGFR. OR, odds ratio; 95% CI, 95% confidence interval.

To further study the associations between the three factors and DR, FT3, RMR and SII were evaluated as quartile variables. As shown in **Table 4**, after rectifying the covariates like age and sex, fourth quartile of FT3 showed significantly decreased OR of 0.615 (95% CI: 0.380 to 0.996; p =0.048) while SII showed increased OR of 1.660 (95% CI: 1.061 to 2.597, p=0.026) for DR with respect to their first quartile value. In the fully adjusted models, fourth quartile of RMR showed significantly decreased OR of 0.291 (95% CI: 0.099 to 0.856; p =0.025) for DR with respect to its first quartile value. However, there were no significant differences between the quartile 1–4 of SII and FT3 and DR in the fully adjusted models.

**Table 4:** Multivariate logistic regression analysis of the independent factors for presence of DR (FT3, RMR and SII as categorical variable).

Variable	Model 1		Model 2		Model 3	
	OR (95%CI)	P value	OR (95%CI)	P value	OR (95%CI)	P value
FT3						
quartile 1	Reference		Reference		Reference	
quartile 2	0.680(0.441-1.048)	0.080	0.710(0.458-1.099)	0.125	0.667(0.414-1.074)	0.096
quartile 3	0.726(0.472-1.115)	0.144	0.778(0.498-1.216)	0.271	0.851(0.526-1.377)	0.511
quartile 4	0.553(0.353-0.866)	0.010*	0.615(0.380-0.996)	0.048*	0.718(0.427-1.208)	0.212
P for trend		0.016*		0.076		0.358
RMR						
quartile 1	Reference		Reference		Reference	
quartile 2	0.855(0.558-1.312)	0.474	0.663(0.377-1.164)	0.152	0.554(0.285-1.076)	0.081
quartile 3	0.710(0.457-1.102)	0.126	0.497(0.243-1.013)	0.054	0.304(0.124-0.743)	0.009*
quartile 4	0.638(0.408-0.999)	0.049*	0.466(0.210-1.035)	0.061	0.291(0.099-0.856)	0.025*
P for trend		0.033*		0.076		0.03*
SII						
quartile 1	Reference		Reference		Reference	
quartile 2	0.915(0.567-1.475)	0.715	0.907(0.562-1.465)	0.690	0.835(0.500-1.393)	0.490
quartile 3	1.489(0.949-2.336)	0.083	1.450(0.922-2.280)	0.108	1.258 (0.774-2.044)	0.354
quartile 4	1.737(1.115-2.708)	0.015*	1.660(1.061-2.597)	0.026*	1.294(0.796-2.104)	0.298
P for trend		0.003*		0.006*		0.127

FT3 quartile 1, 3.53-4.51; quartile 2, 4.52-4.91; quartile 3, 4.92-5.39; quartile 4, 5.40-7.37.

RMR quartile 1, 840.25-1236.50; quartile 2, 1236.51-1493.75; quartile 3, 1493.76-1658.75; quartile 4, 1658.76-2218.75.

SII quartile 1, 78.92-317.67; quartile 2, 317.68-430.57; quartile 3, 430.58-595.58; quartile 4, 595.59-2810.37.

Model 1, not adjusted for any variable; Model 2, adjusted for age, sex; Model 3, adjusted for model 2, BMI, duration of diabetes, SBP, HbA1c, LDL-C, ALT and eGFR. OR, odds ratio; 95% CI, 95% confidence interval.

*Statistically significant (p < 0.05).

## Discussion

Diabetic retinopathy is a devastating ocular impediment and it is considered as the leading cause of new-onset blindness globally. Unless substantial improvements occur in the prevention and treatment of DR, the prevalence and burden of DR will continue to escalate as the global population ages and the epidemic of DM expands (26). Hence it is pressing to explore the specific and sensitive biomarkers to diagnose and detect early disease (2). To the best of our knowledge, we were the first study to investigate the association of TH, RMR and SII with DR in Chinese T2DM patients with euthyroidism, which indicated that lower FT3, RMR and higher SII levels were associated with the presence of DR.

The pathophysiology of DR is extremely complex and multifactorial, and remains elusive. To date, more and more evidence points out that insulin resistance (27), chronic low-grade inflammation (28) and oxidative stress (29) contribute to the occurrence and development of DR. Among these factors, chronic low-grade inflammation plays key role in the pathogenesis of DR (30).

Leukocyte exudation is the most important feature of the inflammatory reaction (30). The SII provides information about multiple immune cells, including neutrophils, platelets, and lymphocytes, and is increasingly regarded as a reliable biomarker of inflammation (31). So far the conclusions about the relationship between SII and DR are somewhat inconsistent. Wang et al. (30) and Li et al. (21) found that SII levels in the DR group were higher than those in the NDR group. While in study performed by Dascalu et al. (23) significantly higher values of SII were only found in the PDR group when compared with NDR and NPDR groups, and no differences were noticed between the latter two groups. Gao et al. (22) confirmed that SII levels tented to increase with the severity of DR, which might reflect the progression of the DR. In agreement with the conclusion by Wang et al. and Li et al. (22), we also found obviously elevated SII in DR, however, we did not further compare the indicator based on the grade of DR.

RMR provides an estimate of the minimum amount of energy expenditure under resting conditions. Recently, Roudi et al. reported that an increased RMR/Weight decreased the likelihood of developing metabolic syndrome (32). Nevertheless, clinical research about RMR and DR are extremely limited. Sasongko et al. showed that RMR was strongly associated with and contributed considerably to the presence and severity of DR (12). Quite the reverse, our research showed that RMR protected against DR in Chinese euthyroid patients with T2DM. In our study, RMR was positively correlated with BMI but negatively correlated with SII. Possible explanations for the conclusions were as follows. Although BMI is a principle factor of obesity, it cannot distinguish body composition including fat, muscle, and bone tissue. RMR is positively correlated with fat-free mass and largely determined by the most metabolically active tissues, for example, skeletal muscle, heart, brain, kidney, and liver (33). Because of its size, skeletal muscle mass is the major correlate of RMR (33). Skeletal muscle can be regarded as an endocrine organ that can release a variety of myokines, which can exhibit anti-inflammation effect (34). Prior study indicated that resistance exercise training reversed skeletal muscle strength, enhanced RMR while improving systemic inflammation (35). Furthermore, higher RMR is positively related with better mitochondrial function (33), which is clearly master regulators of inflammation (36). Therefore, in non-obese individuals with similar BMI, a higher RMR may represent more muscle mass and better mitochondrial function, both of which have anti-inflammatory effects. In addition, previous study has found that FT3 positively correlates with skeletal muscle mass in euthyroid individuals (37). Our data extend this by demonstrating a positive correlation between FT3 and RMR, suggesting that relatively lower FT3 levels in the DR cohort may partially mediate the relationship between reduced RMR and increased DR risk.

TH play important role in maintaining retinal functional homeostasis. However, clinical research about TH and DR are somewhat contradictory. Some studies found a correlation between DR and FT3 while excluding any significant association with FT4(10). Conversely, other studies suggested that DR is exclusively linked to FT4 while downplaying the role of FT3(11). In this study, we only found that circulating FT3 levels was negatively correlated with the incidence of DR in T2DM patients with euthyrodism, no substantial disparity has been observed in the prevalence of DR with FT4. Recent conclusions from basic research provide potential theoretical basis for this discovery. *In vivo* or *in vitro* exposure of retinal cells to elevated glucose concentrations resulted in significant downregulation of type 2 deiodinase 2 (DIO2) (38), the enzyme responsible for the activation of T4 into T3, paralleled by upregulation of type 3 deiodinase (DIO3) (39), a fetal protein which converts T4 and T3 into the biologically inactive reverse T3 (rT3) and T2, eventually leading to a significantly decreased T3 and increased T4/T3.

So far mechanisms underlying the effect of T3 on retinopathy have been little investigated. The study by Bapputty et al. demonstrated that T3 prevented the high glucose induced rise in endothelial nitric oxide synthase, intercellular cell adhesion molecule 1, thus inhibiting oxidative stress and inflammatory response (38). Another study by Forini et al. indicated that low T3 state (LT3S) affect the retinal response to stress through an action on mitochondrial function, which are characterized by early and transient activation followed by later decline (39). A LT3S in early phases seems to have beneficial effects, however, during the chronic phase it becomes detrimental, manifesting as increased oxidative stress, inflammation, VEGF expression, and BRB damage. Moreover, T3 could protect retina from high glucose-mediated cell death (38). Our research indicates that T3 was correlated with RMR, but not with SII. This may be attributed to its anti-inflammatory and pro-inflammatory effects at different stages of DR, suggesting that T3 may not solely protect the retina through its inflammatory regulatory properties. The intrinsic mechanism between T3 and DR required further in-depth research.

In the present study, DR was found to be associated with insulin use. For one, insulin treatment often reflects endogenous insulin insufficiency in T2DM. Endogenous insulin plays an important role in the prevention of DR by retinal insulin receptor-PI3K signaling pathway, that is not achieved by exogenous insulin alone (40). For another, insulin deficiency is commonly associated with poor glycemic control, which is itself a risk factor for DR (40). Furthermore, we found that fasting C-peptide levels protected patients from DR, which was consistent with previous study of Huang et al. (41). In conditions of glucolipotoxicity, C-peptide increases catalase expression and reduces peroxisomal oxidative stress and death of β cells (42). Moon et al. (43) found that intravitreal injection of ultra-long-acting human C-peptide in diabetic mice could delay the progression of PDR by reducing hyperglycemia-induced retinal neovascularization, normalizing oxidative stress, vascular leakage, and inflammation and restoring blood-retinal barrier function. In addition, we found that total bilirubin was negatively correlated with the incidence of DR. Similar results were reported by Ding et al. (44) and Kudo et al. (45). Bilirubin was previously considered to be metabolic waste, however, it has recently regarded as an important strong antioxidant and cytoprotectant.

Also in the present study, we have found that DR patients exhibited decrease ALT, AST and eGFR. Reduced eGFR might indicate the concurrent presence of diabetic kidney disease, which shares a common microvascular pathophysiology with DR (46). However, recent research on the correlation between ALT, AST and DR yielded contradictory conclusions. In line with our study, a prospective cohort study by Deravi et al. demonstrated that ALT and AST were also inversely correlated with the incidence of DR (47). Further studies are essential to investigate the underlying biological mechanisms.

There are some limitations in this study. First, this is a cross-sectional study which could not get the conclusion of cause-and-effect relationship between study indicators and DR. Secondly, due to constraints in the original study design and limited sample size, we were unable to stratify data by DR severity (non-proliferative vs. proliferative). Whether these parameters associate with DR progression remains to be determined. Third, as a one-center study conducted in Chinese subjects, it is unclear whether the findings are applicable to individuals of other ethnicities or in other countries. Therefore, multi-center and multi-ethnic studies should be performed to validate conclusions’ reproducibility. And finally, the diagnostic accuracy of both individual markers and their combination is not high enough for clinical application. These findings highlight the importance of future studies integrating additional parameters, such as metabolic, inflammatory or imaging biomarkers, to develop more robust predictive models.

## Conclusions

In conclusion, decreased FT3, RMR and increased SII were correlated with the presence of DR in T2DM patients with euthyroidism. Although higher FT3 and RMR levels are protective against DR, the limited diagnostic efficacy of these parameters suggests that multi-parameter models or the identification of additional biomarkers are needed to improve the accuracy of early DR screening.

## Data Availability

The raw data supporting the conclusions of this article will be made available by the authors, without undue reservation.
